# A Facile Strategy to Prepare Small Water Clusters via Interacting with Functional Molecules

**DOI:** 10.3390/ijms22158250

**Published:** 2021-07-31

**Authors:** Shanmeiyu Zhang, Yanyan Zhang, Chongchong Wu, Hui Yang, Qiqi Zhang, Fuyi Wang, Jingyi Wang, Ian Gates, Jinben Wang

**Affiliations:** 1 CAS Key Lab of Colloid, Interface and Chemical Thermodynamics, Institute of Chemistry, Chinese Academy of Sciences, Beijing 100190, China; zhangsmy@iccas.ac.cn (S.Z.); zhangqq_bift@163.com (Q.Z.); jbwang@iccas.ac.cn (J.W.); 2 Beijing National Laboratory for Molecular Sciences, CAS Key Laboratory of Analytical Chemistry for Living Biosystems, Institute of Chemistry, Chinese Academy of Sciences, Beijing 100190, China; zhangyy0816@iccas.ac.cn; 3 Department of Chemical and Petroleum Engineering, University of Calgary, Calgary, AB T2N-1N4, Canada; chongchong.wu@ucalgary.ca (C.W.); jwang@ucalgary.ca (J.W.); idgates@ucalgary.ca (I.G.); 4 University of Chinese Academy of Sciences, Beijing 100049, China

**Keywords:** water cluster, functional molecule, molecular interaction

## Abstract

Although small water clusters (SWCs) are important in many research fields, efficient methods of preparing SWCs are still rarely reported, which is mainly due to the lack of related materials and understanding of the molecular interaction mechanisms. In this study, a series of functional molecules were added in water to obtain small water cluster systems. The decreasing rate of the half-peak width in a sodium dodecyl sulfate (SDS)–water system reaches ≈20% at 0.05 mM from ^17^O nuclear magnetic resonance (NMR) results. Based on density functional theory (DFT) and molecular dynamics (MD) simulation calculation, it can be concluded that functional molecules with stronger negative electrostatic potential (ESP) and higher hydrophilicity have a stronger ability to destroy big water clusters. Notably, the concentrations of our selected molecule systems are one to two magnitudes lower than that of previous reports. This study provides a promising way to optimize aqueous systems in various fields such as oilfield development, protein stability, and metal anti-corrosion.

## 1. Introduction

Water clusters are widely studied for their central role in life, geophysics, biochemistry, etc. [1,2,3,4]. The clusters assemble or aggregate from single water molecules via a discrete hydrogen bond (HB), over a wide range of (H_2_O)*_n_*_=2~28_ evaluated by experimental and theoretical approaches [5,6,7,8,9,10,11,12]. The lifespan of water clusters in liquid water is extremely short of a few picoseconds, and hydrogen bonds can continuously form and disappear [13]. Therefore, one water molecule participates in the formation and disappearance of multiple water clusters at a certain moment, leaving a grand challenge in preparing small water clusters in a liquid water system. Studies on small water clusters (SWCs) are not only fundamentally important for revealing the quantum dynamics in water droplet but also practically valuable for applying in various areas, such as conformations of biological macromolecules (particularly proteins and nucleic acids), suitable waterflooding in ultralow permeability reservoirs, and the dissolution of ions in minerals [14,15,16,17,18,19].

Physical methods, such as the methods of magnetic field, electric field [20], temperature [21], and microwave radiation, can reduce the size of water clusters to some extent, but it is still a challenge without external physical fields. Therefore, the effect of chemical additive imposing on the structure of SWCs has attracted tremendous attention [22,23,24,25,26]. For example, cations could increase the median water cluster size in common electrolyte aqueous solution systems and anions decrease it, as revealed by the half-width of ^17^O nuclear magnetic resonance (NMR). The radius of ions with equal ionic charge is bigger, and the effect of the ions caused is stronger, as in the following order: Na^+^ <K^+^ < Mg^2+^ < Ca^2+^ < Al^3+^ < Fe^3+^ and CO_3_^2−^ < SO_4_^2−^ [22,23]. Similarly, halide anions with a larger ion size could influence the long-range HB network and the local hydration number of anions at a great degree through the measurement of liquid time-of-flight secondary ion mass spectrometry (SIMS) [24]. The hydrophobic group of sodium dodecyl sulfate (SDS) molecules was considered to cut off some HBs in the formation of more highly active water clusters; it is shown in fluorescence studies that the ligand can form between water molecules and SDS molecules in aqueous solution [25]. The addition of sodium formate (SF) is able to influence the network structure of water HB from computational chemistry simulations, presumably promoting the growth rate of the water clusters [26]. Although the addition of chemical molecules suggests a promising way to break the HB network of the water system and reduce the size of the water cluster, the type of chemicals and the impact on disassembling water clusters are still limited.

With this in mind, we selected two series of functional molecules with different hydrophobicity or electrostatic potential (ESP) and introduced them into water systems, including the molecules with the same anion head group but different carbon chain lengths, and those with the same carbon chain length but different anion head groups. The half-peak width in ^17^O NMR measurements was used to compare the size of water clusters, and the distribution of water cluster ions H(H_2_O)_n_^+^ was investigated through in situ liquid SIMS measurements, which have been indicated to be a powerful tool to provide direct molecular evidence for the structure of various liquids [27]. Combined with density functional theory (DFT) and molecular dynamics (MD) simulation calculations, the effects of hydrophobicity and ESP of the molecules on destroying big water clusters, as well as the mechanisms of molecular interactions, were revealed. This study aims to introduce a facile way to prepare a small water system via interacting with functional molecules, shedding light on both theoretical and practical fields.

## 2. Results and Discussion

**Figure 1:**
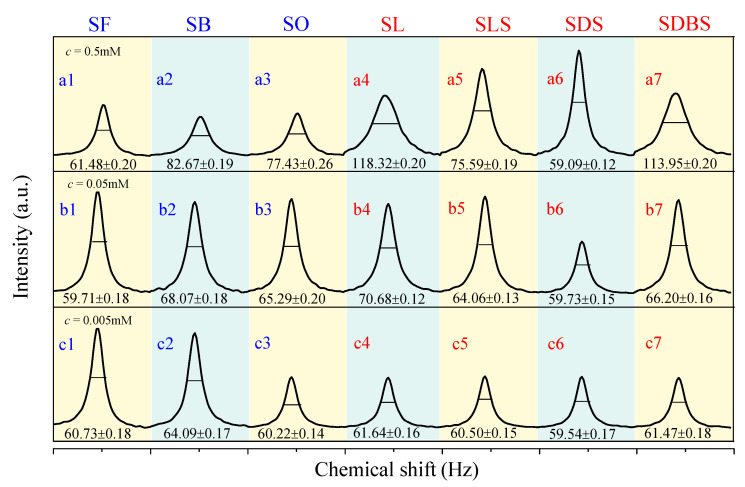
^17^O NMR spectra of water systems including functional molecules at different concentrations of 0.5 mM (**a1**–**a7**), 0.05 mM (**b1**–**b7**), and 0.005 mM (**c1**–**c7**).

The half-peak width of the ^17^O NMR line is used as an index to measure the average relative size of the cluster of liquid water, as shown in Figure 1. The wider the spectral line, the bigger the water cluster, and vice versa [23]. Upon comparing the half-peak width values of these functional molecules to that of pure water (73.98 Hz), we found that the average size of water clusters is smaller in the SDS–water and SF–water system at the series of concentrations (All the abbreviations of functional molecules are defined and listed in Table 1). From 0.5 mM to 0.005 mM, the decreasing ratio of the half-peak width in the SF–water system reaches 16.9–19.3%, and that in the SDS–water system reaches 19.3–20.1%. When the concentration is 0.005 mM, the decreasing of the half-peak width in all the functional molecules reaches the range of 16.9–19.5%. Compared with a previous report [28], the functional molecules in our study can reduce the size of water clusters to a large extent, and the effective concentration of the molecules is 1–2 orders of magnitude lower.

For functional molecules with the same carboxyl head group but different carbon chain lengths, the sequence in the level of decreasing mean water cluster size is: SF > SO (or SB) > SL. The longer the carbon chain length is, the weaker the effect of functional molecules on destroying the big water cluster, which is mainly due to the enhancement of hydrophobic interactions. For those with the same carbon chain length but different anion head groups, the sequence in the level of decreasing mean water cluster size is: SDS > SLS > SDBS > SL. We speculate that the above decreasing sequence is mainly related to the negative ESP of the functional molecules, which will be further discussed in the following.

The ^17^O NMR line-width of the water system in the case of different functional molecules at the concentration of 0.05 mM within 80 days was tested, as shown in Figure S1. In the pure water system, the half-peak width increases 73% after 80 days, indicating that when pure water is placed, the size of water clusters is increasing. The results for SF–water, SB–water, SL–water, SDBS–water, and SO–water systems exhibit that the half-peak width increases 103%, 50%, 41%, 44%, and 20% after 80 days, respectively. In comparison, small water clusters can be formed in the presence of amphiphilic functional molecules, especially in the SLS–water and SDS–water systems, with the decrease in half-peak width of 11.5% and 7.3%. It can be concluded that without hydrophobic groups in the molecules, taking the SF–water system as an example, the half-peak width increases sharply with time, as shown in the functional molecule–water system.

**Figure 2:**
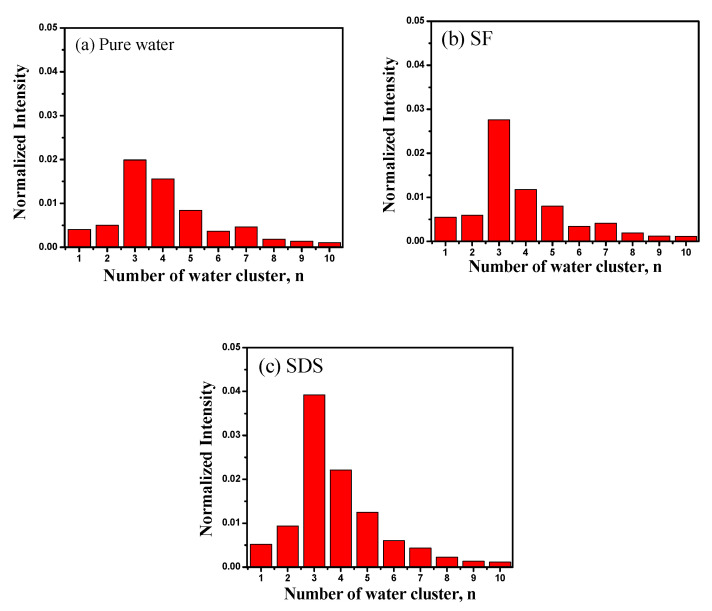
Histograms of normalized cluster ions (*n* = 1–10) in the case of (**a**) pure water, (**b**) SF system at 0.05 mM, and (**c**) SDS system at 0.05 mM.

SF–water and SDS–water systems at 0.05 mM were selected and tested through in situ liquid SIMS measurements, in which the distribution of water clusters can be determined with direct molecular evidence. H(H_2_O)_n_ with *n* = 2–4 are considered to be the main SWC types for the liquid water, in which H(H_2_O)_3_^+^ is the most dominant one [29,30]. It is notable that the percentage of the normalized signal intensity of SWCs (*n* < 5) to the detected water cluster ions (*n* = 1–4) in SF–water and SDS–water systems is 71.9% and 73.4%, respectively, which is higher than 67.8% in the pure water system (Figure 2). The presence of SF and SDS makes the amount of SWCs increase, indicating that these two functional molecules can destroy big water clusters and facilitate the formation of SWCs, supporting the conclusion from the ^17^O NMR results that they can turn big water clusters into small and uniform water clusters. By contrast, halide anions were introduced to pure water, but the total content of SWCs was not significantly increased in comparison with that of pure water [24].

**Figure 3:**
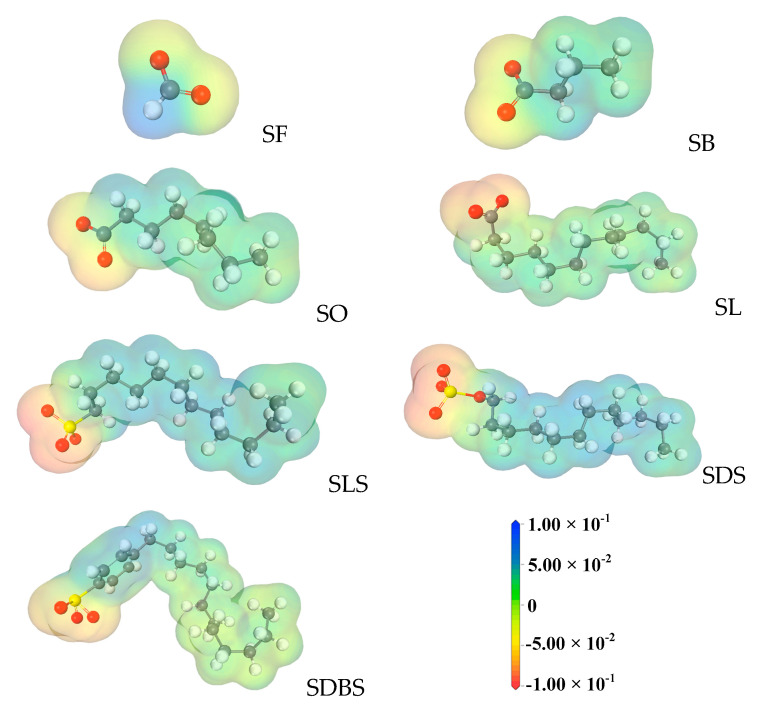
Molecule electrostatic potentials (ESP) of functional molecules selected in this study.

HLB values of the functional molecules evaluated by the method of molecular structure [31] are shown in Table S1, indicating that a bigger HLB value represents stronger hydrophilicity. Among the functional molecules, the sequence of hydrophilicity is SDS > SF > SB > SO > SL > SLS > SDBS. ESP results are shown in Figure 3, where darker color red represents more electrons gathering. Except for the SF molecule, the other functional molecules all have relatively separated positive and negative ESPs due to the long distance between anionic and cationic groups, leading to the electrostatic interaction [32]. Comparing the four functional molecules with the same hydrophobic chain length but different anion head groups, the decreasing order of negative ESP is SDS > SLS > SDB S > SL. The presence of oxygen atoms in the molecular structure is the main prerequisite for negative ESP and, consequently, the sequence of the ESP is determined by the number of oxygen atoms in the head groups. It agrees with the results from NMR experiments that more SWCs can be formed under the regulation of functional molecules with stronger negative ESP. Accordingly, SDS molecules, owning the strongest negative ESP and the strongest hydrophilicity, facilitate the formation of the small water clusters.

**Figure 4:**
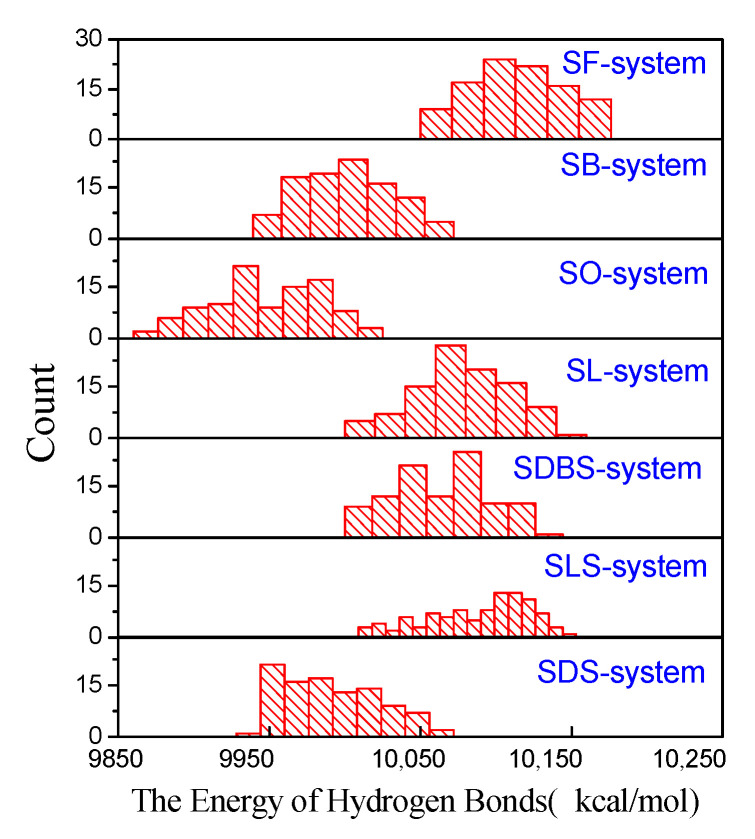
The histograms of energy of the hydrogen bond after adding functional molecules (the y-axis represents the number of times the hydrogen bond energy occurs in the simulation process).

The energy of the hydrogen bond after adding functional molecules is depicted in Figure 4. When adding one functional molecule, the energy of HBs in the system is much lower than that in the pure water system (10,657 kcal/mol in Figure S2). It shows that functional molecules greatly destroy large water clusters and facilitate them into small water clusters or individual water molecules. The lower the energy of HBs, the less the number of HBs in the system, and probably the smaller the average size of the water clusters.

Among functional molecules with the same carboxyl head group but different carbon chain lengths, the SO system shows the lowest energy of HBs and the SF system shows the highest. The simulation calculation results also reveal that there is hardly any HB interaction between the water molecules and the carbon chain. It shows that the functional molecules with a longer carbon chain own a stronger ability to destroy big water clusters, even if the carbon chains do not form HBs with water molecules.

Regarding the ability of functional molecules with the same hydrophobic chain length but different anion head groups to destroy water clusters, SDS is the best. They have the same carbon chain length, and therefore, the degree of water molecules repelling the carbon chain is similar. The oxygen atom with negative ESP forms HB with the hydrogen atom of the water molecule (with positive ESP), so the ability of the head group to break the HB of water clusters is related to its negative ESP. SDS has a strong negative ESP, which is attributed to the SDS system having low energy HBs from simulation calculation.

**Figure 5:**
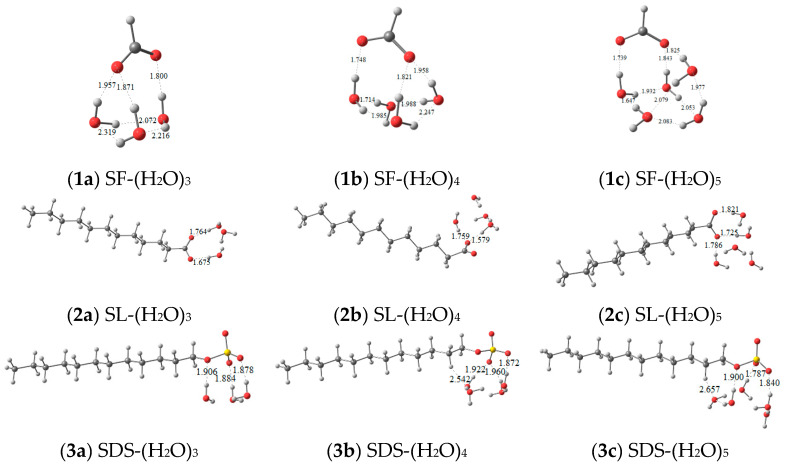
Optimized geometries of (H_2_O)*_n_*_=3–5_ with SF (**1a**–**c**), SL (**2a**–**c**), and SDS (**3a**–**c**).

The optimized geometries between functional molecules and water clusters (H_2_O)_*n*=3–5_ are shown in Figure 5, the initial structures for which were built according to the electrostatic potential analysis of water clusters (Figure S3) and functional molecules (Figure 3). Accordingly, the number and the length of HBs forming between functional molecules and water molecules can be obtained. Hydrogen bonds in water clusters are continuously forming and disappearing [13]; functional molecules interacting with more water molecules, or in a looser interaction with water molecules, probably have a strong impact on destroying pure water HB systems. Three or four HBs form between the interactions of SDS and (H_2_O)_*n*=3,4,5_ more than the interactions of SF or SL and (H_2_O)_*n*=3,4,5._ For functional molecules with the same carboxyl head group but different carbon chain lengths, such as SL and SF, the SL and (H_2_O)_*n*=3,4_ system forms fewer HBs with shorter bond lengths than that of SF–water, indicating that SL interacts strongly with water molecules. The strong HBs that formed between SL and water limit SL molecules from joining in destroying other water clusters. In comparison, the HBs in the SF–water system are long and weak; SF molecules are more flexible in water to form HBs with other water molecules and break the HBs of large water clusters. For functional molecules with the same hydrophobic chain length but different anion head groups, such as SL and SDS, three HBs are formed between (H_2_O)_5_ and SL, and four HBs are formed between (H_2_O)_5_ and SDS. The head group of SDS more easily forms HBs with water molecules than that of SL. It can be concluded that the ability of SDS molecules to interact with water molecules and disassemble the pure water clusters is stronger than that of SL, which is consistent with the results from NMR. Combined with Figure S4, it was found that in a SDS–water system, the HBs in SWCs are relatively short.

## 3. Materials and Methods

Materials. The functional molecules used in this study are shown in Table 1. SF (99.5%) and sodium butyrate (SB; 99.5%) were obtained from Aladdin Chemical Co. Ltd. Sodium octanoate (SO; 98%) was purchased from Adamas-beta (Shanghai, China). Sodium laurate (SL; 99.8%), sodium lauryl sulfonate (SLS; 99.5%), sodium dodecyl sulfate (SDS; 99.8%), and sodium dodecyl benzene sulfonate (SDBS; 99.8%) were purchased from Sinopharm Chemical Reagent Co. Ltd. (Shanghai, China). All the above chemicals were recrystallized three times before use and analyzed through elementary analysis (see Table S2). Deionized water (resistivity = 18.2 MΩ·cm) from Milli-Q equipment was used in all experiments.

The critical micelle concentration (CMC) of four functional molecules including SDS, SL, SLS, and SDBS was obtained by measuring the surface tension, as shown in Figure S5. The concentrations selected in this paper were all below CMC.

Methods. ^17^O NMR. All ^17^O NMR chemical shifts and half width were measured via a Bruker AVⅢ 500WB superconductor spectrometer, which was obtained at 67.786 MHz using a zg pulse. Each free induction decay (FID) had 256 scans with a recycle delay of 0.2s. ^17^O NMR chemical shifts and half width are expressed in Hz (not in the usual ppm) on account of the ultralow concentrations leading to low chemical shifts. The reproducibility of the chemical shifts was better than 2 Hz. The measurements were carried out under 293.2 K (20 °C). The tested concentrations are 0.5 mmol/L (mM), 0.05 mM, and 0.005 mM, respectively.

In situ liquid SIMS. Water systems with a small amount of SF, SL, and SDS, respectively, were tested on a ToF-SIMS V instrument (ION-ToF GmbH, Münster, Germany), which was interfaced with a home-made vacuum-compatible microfluidic cell. The fabrication details of the liquid device as well as the parameters of liquid SIMS experiments were according to our previous method with minor adaptations [27,28]. In brief, a silicon frame (200 µm in thickness) that modifies with a silicon nitride (SiN) membrane (100 nm in thickness) was put on top of a pre-machined liquid chamber (3.0 mm in length, 3.0 mm in width, 0.3 mm in height) on a poly (ether-ether-ketone) block and sealed with glue. Then, a liquid sample was introduced into the liquid chamber through two liquid channels on both sides of the block. Afterwards, we sealed both of the channel ends and loaded the fabricated device into the ToF-SIMS analysis chamber for liquid SIMS analysis. During the measurements, we focused a pulsed 25 keV Bi_3+_ analysis beam to ≈350 nm. The beam pulse width was ≈150 ns, and the beam current at a pulse repeating frequency of 10 kHz was 0.289 pA. The beam was scanned on a round area of ≈2 μm in diameter on the SiN membrane window, the penetration of which was indicated by a sudden increase in the signal intensities of the species in liquids. When the signals of liquids became relatively stable, the beam pulse was narrowed to 50 ns for a relatively higher mass resolution. After collecting signals with reasonable intensities, we could stop the measurements. The pressure in the main chamber during the measurements was 5 × 10^−7^ mbar. Due to the limitation of the surface tension by the test conditions, the proper concentration is selected to be 0.05 mM.

MD simulations. All the molecular dynamics simulations with different modules are computed by Materials Studio 7.0 software. Simulation I was used to calculate the ESP of different functional molecules. The potential energy surface is important in reflecting the aggregation properties of molecules, which is with respect to the presence of water molecules [29]. The Energy task was run along with the ESP in properties by the Dmol3 Calculation. We imported the Potentials and Electron density, respectively, and then selected the electrostatic potential in the mapped field in order to display. The same scale (−0.1~0.1 au) was selected for the scale bar of ESP calculated for different molecules.

The variation value of the HB energy, which resulted from adding functional molecules in the water, was calculated and used to further determine the effect of functional molecules on the energy. The interaction energies of one functional molecule with 200, 2000, or 20000 water molecules, respectively, were calculated, and it was that the energy values of 2000 and 20000 water molecules are very similar, so we selected 2000 water molecules in one interaction for calculation convenience. Simulation II proceeds as follows: (i) The water clusters box (contained 2000 water molecules) and the boxes contained functional molecules, such as SL, SO, SB, and SF, respectively, were all constructed by the Amorphous cell tool; (ii) Both the water clusters box and the box that contained functional molecules were merged to the final cell through building a layered structure as a crystal; (iii) After the optimization of the final box, molecular dynamics simulations were performed, in which the Quench was selected. During the entire simulation process, Dreiding is selected for the force field.

MD simulation experiments with 100 random initial positions were performed. The histograms of energy of the hydrogen bond in every system were obtained, exhibited in Figure 4, and the peak of statistics is attributed to the most stable configuration.

DFT Calculations. The interactions between SDS/SF/SL anion and water clusters were investigated through DFT calculations by using the Gaussian 09 software [30]. We used the second-order Møller–Plesset perturbation (MP2) method and 6-311++g (d,p) basis set, which have been found to be accurate for water clusters calculations [31,32]. The geometries of all water clusters considered here were optimized by using an MP2/6-311++g (d,p) level of theory. The interactions between water clusters and SDS/SF/SL anion were calculated by the same method and basis set. Frequency analyses were conducted to confirm that there is no imaginary frequency that optimizes structures. The ESP analyses were conducted by GaussView 6.0 software.

## 4. Conclusions

Two series of functional molecules were introduced to water cluster systems, which were investigated through the methods of ^17^O NMR, in situ liquid SIMS, MD simulation, and DFT calculation in our study. For functional molecules with the same carboxyl head group but different carbon chain lengths, the ability to break the HBs of water clusters is the same as the molecular hydrophilicity: SF > SB > SO > SL. SF, without the carbon chain and with the strongest hydrophilicity in the series of functional molecules, can interact with more water molecules and destroy big water clusters into small ones. For those with the same hydrophobic chain length but different anion head groups, the ability to break the HBs of water clusters is the same with the negative ESP: SDS > SLS > SDBS > SL, which is in agreement with the conclusions of ^17^O NMR. The decreasing rate of the half-peak width and the amount of H(H_2_O)_3_^+^ in the SDS–water system can reach 20% and 5.6%, respectively, compared with that of the pure water system. It is because SDS with a higher negative ESP and better hydrophilicity can form more HBs with water molecules and break the HBs interior of the water clusters. Furthermore, the HBs among water molecules are relatively short in an SDS–water system, and the ^17^O NMR line-width of the water system remains nearly unchanged for ≈80 days. Our work suggests a general strategy to prepare small water clusters via interacting with functional molecules, providing new ideas to both experimental and computational studies on SWCs.

## Figures and Tables

**Table 1:** Introduction of functional molecules selected in this study.

Name	Molecular Formula	Chemical Formula
Sodium formate (SF)	HCOONa	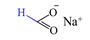
Sodium butyrate (SB)	CH_3_(CH_2_)_2_COONa	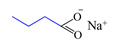
Sodium octanoate (SO)	CH_3_(CH_2_)_6_COONa	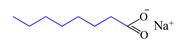
Sodium laurate (SL)	CH_3_(CH_2_)_10_COONa	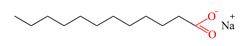
Sodium lauryl sulfonate (SLS)	CH_3_(CH_2_)_10_CH_2_SO_3_Na	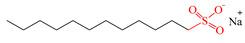
Sodium dodecyl sulfate (SDS)	CH_3_(CH_2_)_10_CH_2_SO_4_Na	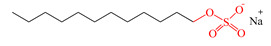
Sodium dodecyl benzene sulfonate (SDBS)	CH_3_(CH_2_)_10_CH_2_C_6_H_4_SO_3_Na	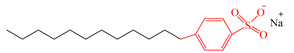

## Data Availability

Not applicable.

## Supplementary Materials

The following are available online at https://www.mdpi.com/article/10.3390/ijms22158250/s1.
