# Supplementary material for: A Facile Strategy to Prepare Small Water Clusters via Interacting with Functional Molecules
Source: Int J Mol Sci. 2021 Jul 31;22(15):8250. doi: 10.3390/ijms22158250 (PMC8347634; doi:10.3390/ijms22158250)
Supplement: Supplementary file 1 [file ijms-22-08250-s001.zip › ijms-1296198-supplementary.pdf]

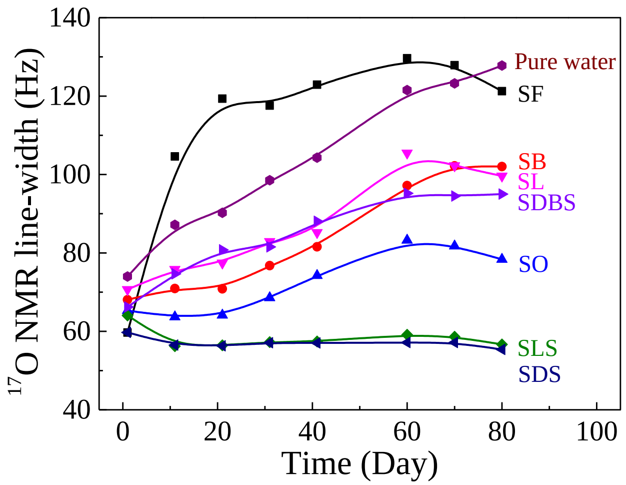

Figure S1. ^{17}O NMR line-width of water system at the concentrations of 0.05 mM with time.

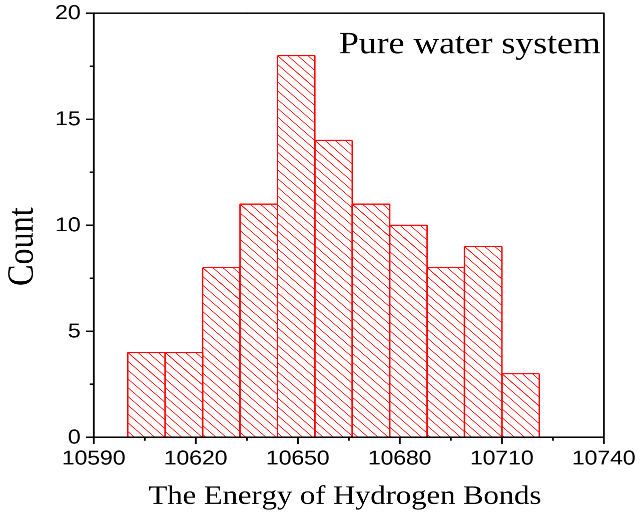

Figure S2. The histograms of energy of hydrogen bonds in pure water system.

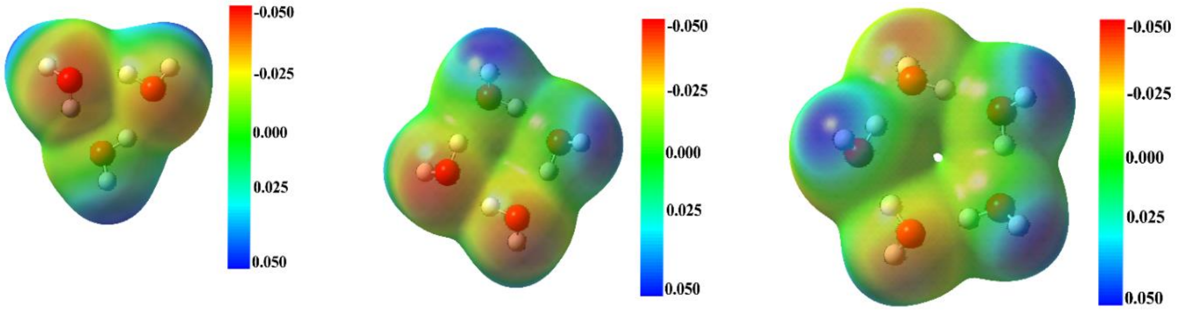

(a) $(H_2O)_3$ (b) $(H_2O)_4$ (c) $(H_2O)_5$

Figure S3. Electrostatic potential analysis of water clusters.

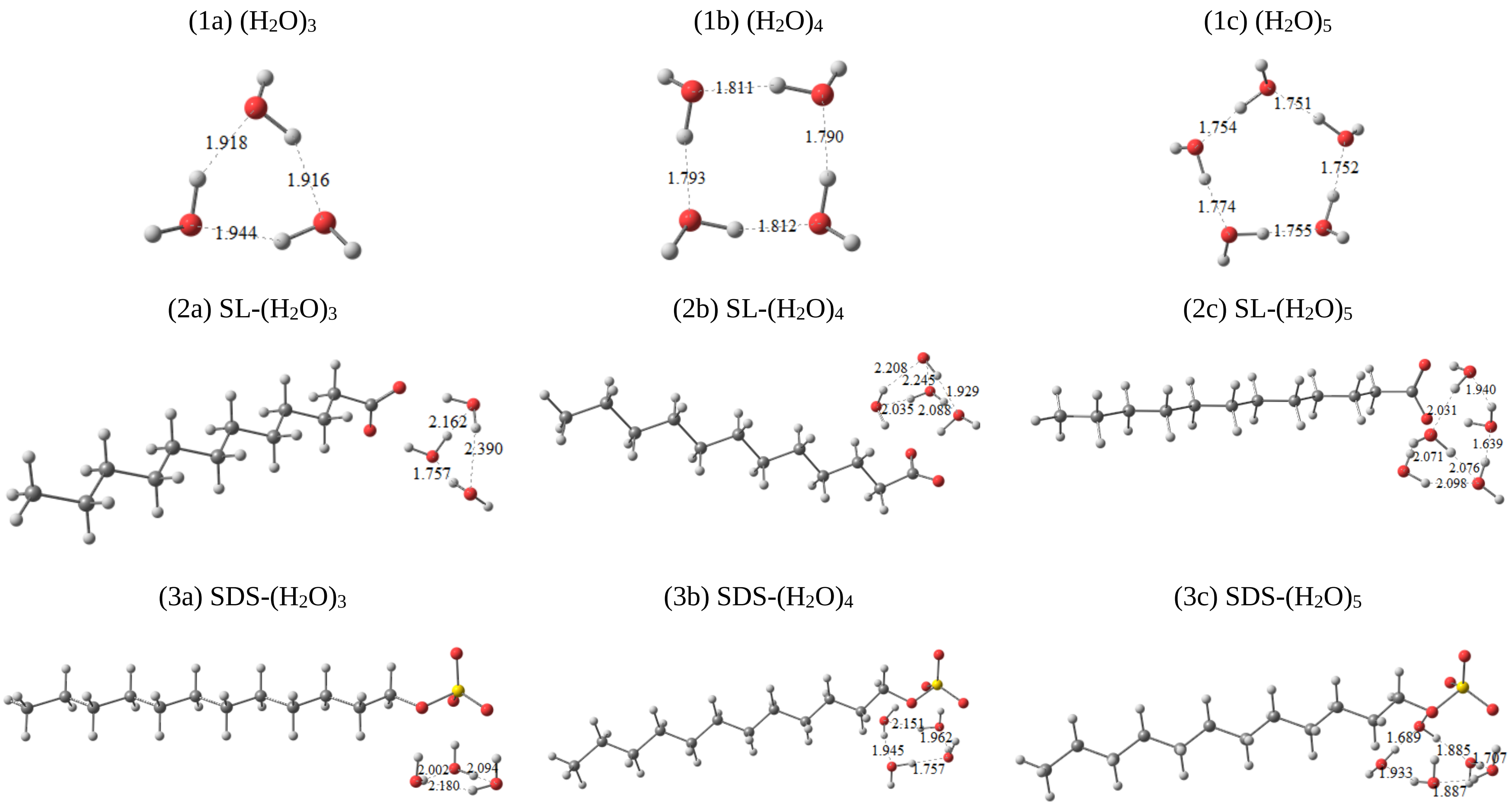

Figure S4. Optimized geometries of $(H_2O)_n$, n=3-5 (1a~c) and $(H_2O)_n$, n=3-5 with SL (2a~c) and SDS (3a~c).

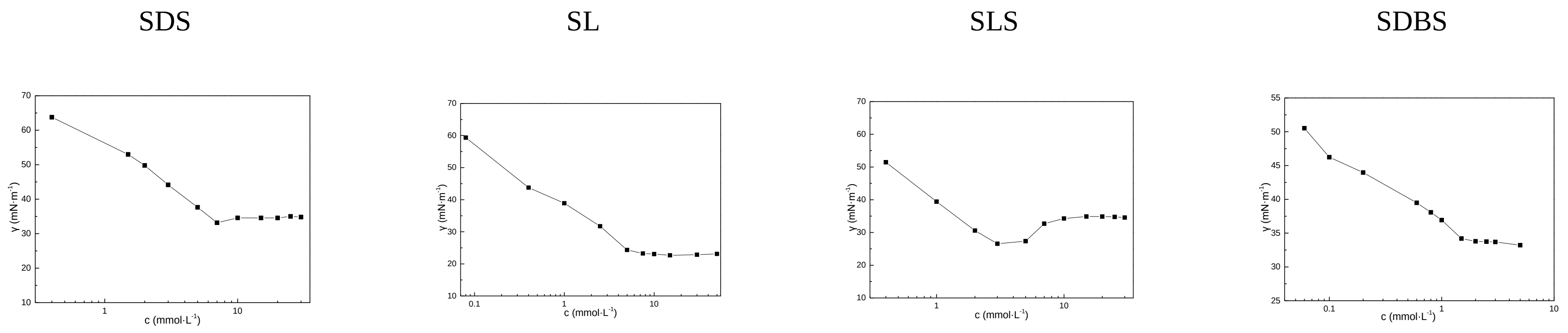

Figure S5. The critical micelle concentration of the four surfactants.

Table S1. HLB value of functional molecules calculated by the method of molecular structure.

Abbreviation	HLB value
SDBS	9.450
SLS	12.300
SL	20.875
SO	22.775
SB	24.675
SF	26.100
SDS	40.000

Table S2. Elemental analysis of functional molecules.

Functional molecule	Elemental analysis (%) Calcd	Elemental analysis (%) Found
**SF**	C, 17.66; H, 1.48	C, 17.71; H, 1.50
**SB**	C, 43.64; H, 6.41	C, 43.68; H, 6.51
**SO**	C, 57.82; H, 9.10	C, 57.86; H, 9.30
**SL**	C, 64.84; H, 10.43	C, 64.99; H, 10.51
**SLS**	C, 52.92; H, 9.25; S, 11.77	C, 53.05; H, 9.31; S, 11.85
**SDS**	C, 49.98; H, 8.74; S, 11.12	C, 50.06; H, 8.82; S, 11.22
**SDBS**	C, 62.04; H, 8.39; S, 9.20	C, 62.11; H, 8.45; S, 9.31
